# Moult Timing as a Measure of Condition in a Northern Hemisphere Ungulate

**DOI:** 10.1002/ece3.74493

**Published:** 2026-11-01

**Authors:** Devyn J. Simeoni, Kirsty H. Macphie, Fiona E. Guinness, Sean J. Morris, Alison Morris, Ziyi Wang, Tim H. Clutton‐Brock, Josephine M. Pemberton

**Affiliations:** ^1^ Institute of Ecology and Evolution University of Edinburgh Edinburgh UK; ^2^ Large Animal Research Group, Department of Zoology University of Cambridge Cambridge UK

**Keywords:** condition, fecundity, moult, parasites, phenology, red deer, reproduction, survival

## Abstract

Many vertebrates that live in seasonal climates moult once or twice a year, with the effect of refreshing worn coat or plumage, increasing insulation or improving camouflage. The timing of moult may be in response to a variety of cues, but because it is costly, it is also likely to reflect the health and wellbeing of individuals, here referred to as their condition. Using the individually‐monitored red deer from the Isle of Rum, in which summer and winter coats are easily distinguished, we (a) test for traits that are predictive of moult timing, (b) explore traits that moult timing is predictive of and (c) analyse covariances between moult timings and calving dates at the individual, annual and residual level. We show that adult female moult timing in July and November is associated with age, reproductive status and parasite burden, and has been getting earlier over time in November. A female's moult timing also predicts the overwinter survival of herself and her current calf as well as her future fecundity. Moult timing varies among individuals and years. Covariances between calving dates and the timing of each moult shed light on the temporal patterns of condition within individuals and across years, for example, across years, winter moult covaries with the following calving date but not the summer moult that occurs shortly after. The timing of moult appears to be a non‐invasive measure of condition. Monitoring moult timing across years at the population level may shed light on year‐to‐year variation in population health and variation within populations is likely indicative of the relative conditions among individuals. Increased knowledge of population and individual condition may be of benefit for ungulate population management and conservation.

## Graphical abstract

It can be hard to directly monitor the health and wellbeing (or condition) of individuals in wild systems, though the information is of high value in population management, conservation and ecological research. We find that the timing of moult in red deer is predicted by energetically costly events and predicts future reproduction and survival. We therefore suggest moult timing could serve as a proxy for individual condition, providing valuable information on average population‐level condition across years and variation among individuals within years.

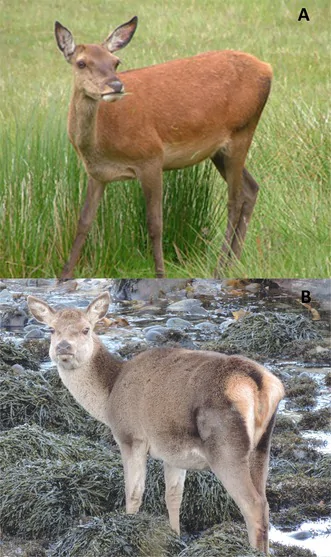

## 1 Introduction

Measuring the health and well‐being of wild animals is valuable but difficult. Vital rates, including survival and reproduction, underpin population dynamics both in endangered populations that require support and in abundant species where management such as culling is required (Beguin et al. 2016; Hutchings 2021; Johnson et al. 2010). Vital rates depend on the health and well‐being of individuals within a population, hereon referred to as individual condition. Condition represents the energetic resources available to individuals that enable them to feed, reproduce and persist in challenging environments, making it a valuable indicator for management and conservation (Bright Ross et al. 2021). However, many wild animals are hard to catch to measure condition via phenotypes such as mass (Santos et al. 2020). As a result, predicting which individuals are most likely to survive or reproduce depending on condition remains an obstacle in ecological research and wildlife management and conservation (Sanz‐Aguilar et al. 2015). Finding a metric of condition that can be observed in the field would be highly advantageous.

Moulting is a variable trait that could serve as a measure of condition for mammals and birds (Beltran et al. 2018; Clutton‐Brock et al. 1982). Here we focus on moulting as a process by which an organism sheds old parts of their body including fur, feathers, or skin cells, to grow new ones between seasons (Mills et al. 2024). In mammals, moulting often occurs biannually and serves to adjust thermoregulation and camouflage (Beltran et al. 2018). The timing of moulting is primarily influenced by changes in photoperiod via hormones (Zimova et al. 2018), modified by other factors as discussed below. While moulting is a widely‐recognised process, there are relatively few studies that investigate moult timing in wild mammals (Beltran et al. 2018; Déry et al. 2019). One of the most well studied wild species is the snowshoe hare ( *Lepus americanus* ), for which the seasonal changes in coat colour are important for camouflage, with survival consequences of mismatches to the environment (Zimova et al. 2018). For many species, however, the benefits and consequences of moult phenology are less heavily associated with camouflage, or often not at all. Moulting is energetically costly, and evidence suggests that an individual in poor condition may prioritise other costly processes including reproduction, thus modifying moult timing (Constant et al. 2024; Déry et al. 2019; Dietz et al. 2013; Larue et al. 2022). Individual‐based studies provide the opportunity to better understand the relationship between vital rates and moult timing.

If moult timing does reflect an individual's condition, then we could expect it to be predicted by prior energetically costly events. There are numerous variables that could predict moult timing, including reproduction, age, parasitism and environmental variation. Reproduction is energetically costly and is often prioritised in energy allocation, which may in turn delay moult timing. For instance, in the Ross Sea, Weddell seal ( *Leptonychotes weddellii* ) moulting is not only influenced by ice break‐out date but is also delayed when reproduction is successful (Beltran et al. 2019). Mountain goat females ( *Oreamnos americanus* ) moult earlier if heavier than expected for their age and if they have not given birth in the same year (Déry et al. 2019). Age may also predict moult timing from early life, through prime, to senescence. Senescence can manifest as lower energy levels, making age a factor in delayed moult timing (de Kock et al. 2021; Déry et al. 2019). Parasitism could also influence moult timing as individuals with a higher parasite load may have less energy to allocate to moulting early. For example, juvenile European starlings ( *Sturnus vulgaris* ) showed delayed moulting when ectoparasites were present in their nests, potentially indicating a fitness cost (Pirrello et al. 2015). Environmental variation may also affect moult timing. For example, in years or locations with higher plant growth, herbivores have access to more forage that may increase available energy to allocate to processes including moulting and reproduction (Jeong et al. 2024; Loe et al. 2005; Macphie et al. 2025). If environmental variation contributes to moult timing, we would expect to see year to year variation in moult timing. In addition to annual variance, long‐term changes in the forage supply, associated with ongoing climatic change, may drive a directional trend in the timing of moult, as observed in other phenological traits (Cohen et al. 2018; Parmesan and Yohe 2003; Romano et al. 2023). If there are genetic or permanent environment effects, the latter perhaps set up by early life environment effects, we would also expect to see variance at the level of the individual.

While moult timing may be predicted by prior energetic events, we might also expect moult timing to correlate with future events that are impacted by individual condition. Body mass is a common indicator of condition, and in white‐tailed deer ( *Odocoileus* virginianus), individuals that are heavier than expected have a higher probability of overwinter survival (Kautz et al. 2020). Similarly, female Alaskan moose ( *Alces alces* ) with thicker rump fat are more likely to become pregnant and have heavier calves than thinner females (Keech et al. 2000). If moult timing is a measure of condition in a similar manner to body mass, then moult timing could also be predictive of future investment in pregnancy, offspring birth weight, offspring survival, or adult survival. Moreover, if earlier moult timing implies higher individual condition, earlier moult timing at the population level could indicate future population growth (Stopher et al. 2014). In mammals, variation in moult timing could offer a valuable management tool, serving as a visible marker for identifying individuals to protect or cull based on an individual's moult status at specific dates.

If the timing of different phenological traits is influenced by individual condition, we may expect to see covariances between phenological traits. Covariances may occur at different levels between different trait pairs, shedding light on how traits at different points in time are connected. Covariance may occur among years, individuals, and at the individual:year level (Clements et al. 2011). Covariance due to inter‐annual variation would indicate population‐level effects in which conditions within a year affect traits and individuals in a similar way (Gilbert et al. 2020). Individual‐level covariance would indicate consistent differences between individuals, perhaps caused by permanent environmental effects or genetic differences (Wilson and Nussey 2010; Clements et al. 2011). Covariance at the individual: year, or residual, level would reflect a pattern in the deviations from annual and individual effects between traits. Investigating the levels at which different phenological traits covary would provide insight into the processes underlying and linking them. For example, it has been suspected that individual condition influences reproductive timing in red deer (Clutton‐Brock et al. 1982); the levels at which different moulting events covary with reproductive timing have the potential to provide valuable information on the time periods that are most influential on individual condition.

Here, we studied moult timing in adult female red deer ( *Cervus elaphus* ) from the Isle of Rum, Scotland, using individual‐level data collected over 37 years (Pemberton et al. 2022). Red deer undergo a biannual moult; changing from a thick greyish coat (Figure 1B) to a thin bright chestnut coat (Figure 1A) in late spring and *vice versa* in autumn (Ryder and Kay 1973; Ryder 1977). Each year, we recorded the moult status of deer through two moult censuses, on 1st July and 1st November (Figure 2). Previous research in the study population suggests moulting may serve as a measure of condition. Females that lactate to a calf over summer are less likely to moult into winter coat by November 1st compared to females whose calves die neonatally (Clutton‐Brock et al. 1989). Individual winter moult timing and calving dates are repeatable and influenced by both genetics and permanent environment effects; however, previous work did not identify a clear covariance between winter moult timing and calving dates at the phenotypic or genetic level (Clements et al. 2011). Now, with double the sample size and data from both summer and winter moult censuses, we return to moult timing with the aim of understanding its relation to individual condition as reflected through various events that occur in each annual cycle (Figure 2).

**FIGURE 1:**
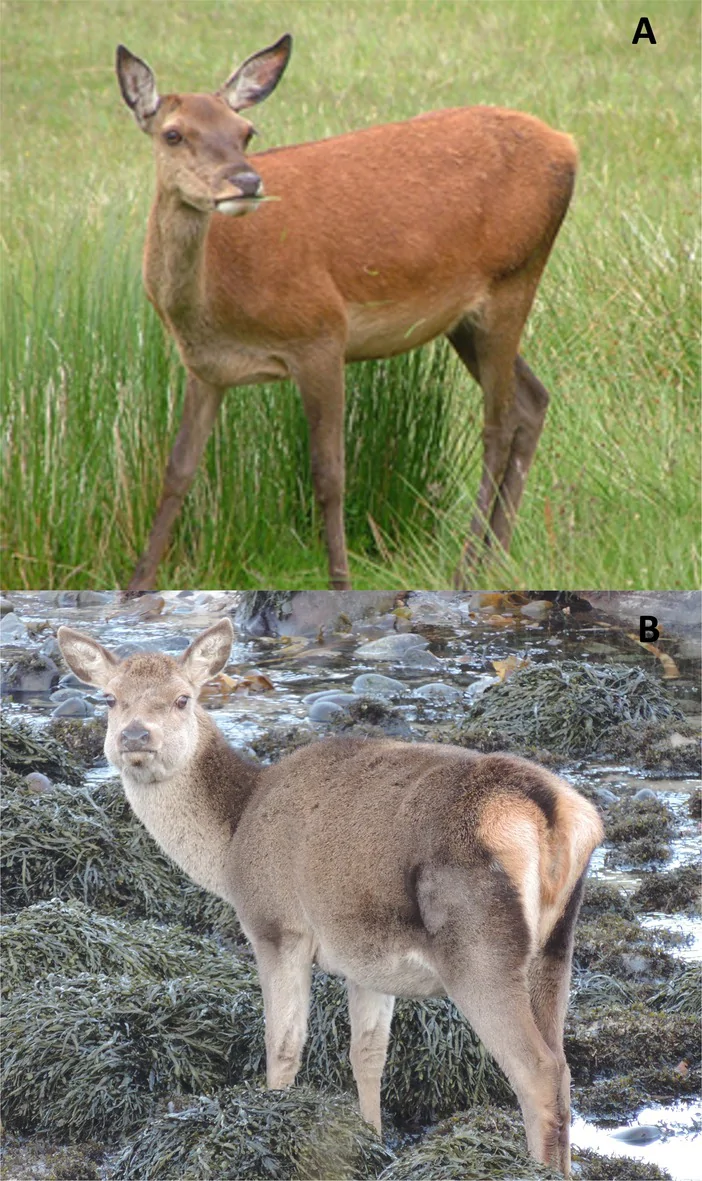
Photos of female red deer in (A) summer coat and (B) winter coat. Photos from Alison Morris.

**FIGURE 2:**
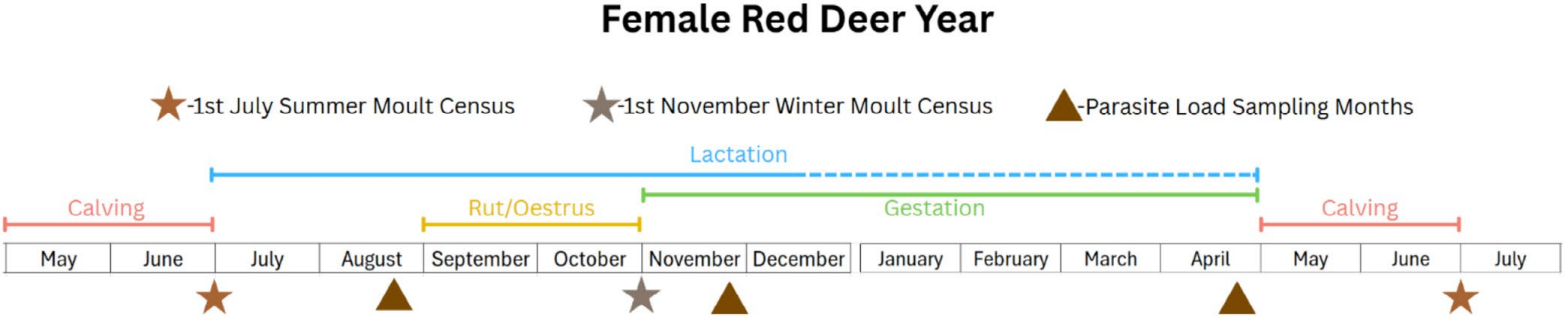
The reproductive cycle of female red deer over a 15‐month period. Each bar colour represents a reproductive stage during the year. Lactation (blue) ends in a dashed line to indicate that some calves are weaned throughout the winter, especially when a mother is pregnant again. The stars represent when each moult census was recorded and the triangles represent parasite sampling sessions.

Specifically, we investigated three aims. First, we examined whether variables that are expected to impact individual condition, including reproductive status, age and parasite load, are predictive of moult status during the moult censuses, and whether there has been any change in moult status over time. Second, we examined whether moult status predicts fecundity, female survival, offspring survival and offspring birth weight; variables that we would expect to be impacted by individual condition. Lastly, we examined the covariances between calving dates and moult status during the summer and winter censuses at the year, individual and residual level. Overall, these aims will help us to determine whether moult status reflects individual condition.

## 2 Methods

### 2.1 Study Site

The data were collected in the North block of the Isle of Rum, Scotland, (N57°, W6°20) where the population of wild red deer has been individually‐monitored since the early 1970s (Clutton‐Brock et al. 1991). In brief, females are observed daily during the calving season to record calving dates. Calves are caught soon after birth, weighed and marked. Individuals are followed for the rest of their lives by regular censusing, through all reproductive events until death, which is natural or by shooting on the fringes of the study area. Birth weights were adjusted for age at capture as birth weight = capture weight – (age in hours*0.01539). Individuals not caught at birth are monitored using distinctive natural features, or a few have been darted for marking.

The moult status of each female was recorded twice a year. On 1st July and 1st November each year a moult census was conducted. We recorded the moult status of each individual resident within the area, categorised as ‘summer’ if more than half of the body was in summer coat, ‘winter’ if more than half of the body was in winter coat and ‘half’ if they were half moulted. Around the census dates *ad hoc* observations of moult status were also recorded. If a deer was not observed on the census date but had been observed fully moulted prior to the census or not yet half moulted after the census, then their moult status on the census date was imputed. We limited the moult census data to adult (2+ years) females which are the most abundant sex‐age class in the study area and show the clearest coat changes. The data for the summer moult censuses were collected between 2001 and 2024, with a sample size of 660 females and 3606 observations. The winter moult data were collected between 1987 and 2023, with a sample size of 905 females and 5579 observations. Sample sizes vary among models due to missing data in other variables.

The other variables used in this study included each female's ID, age in years, reproductive status at relevant time points (see below), calving date (ordinal date from January 1st), calf birth weight, fecundity, female annual survival and calf first year survival, both defined by survival to 1st May the following year. Fecundity was recorded as a binary (0/1) variable based on whether or not an individual had a calf that year.

Reproductive status describes a female's calving and rearing history relative to the given summer or winter moult census. There were five categories for reproductive status: ‘Immature’ means the female had never had a calf before; ‘True Yeld’ means the female had previously given birth but not in the focal year; ‘Summer Yeld’ means the female had a calf in the focal year, but the calf died before 1st October in that year (surviving less than 4 months); ‘Winter Yeld’ means the female had a calf but it died between the 1st October in the year of birth and the 1st May in the next year (surviving less than one year); and ‘Milk’ means that the female had a calf and the calf survived until the 1st May in the year following birth (survived the first year), or the 1st October when used as a predictor of the winter moult census (survived the first four months). In the sections below, we specify how reproductive status varies in each model.

Parasite load was recorded using faecal egg counts between 2016 and 2024. Three times a year, in April, August and November, faecal samples were collected from known individuals and processed to quantify strongyle faecal egg counts (FECs) in eggs per gram (full method details in Albery et al. 2018). Strongyles are a group of gastrointestinal nematodes that infect ruminants and have a direct life cycle. High FECs are associated with depressed fecundity and juvenile survival in the Rum deer (Acerini et al. 2022; Albery et al. 2021).

### 2.2 Statistical Analysis

All analyses were run using Markov Chain Monte Carlo (MCMC) generalised linear mixed models (GLMMs) in the MCMCglmm package in R (Hadfield 2010; R Core Team 2025). Models and model specific sample sizes for each analysis are outlined in Supporting Information.

### 2.3 Aim 1: What Predicts Moult Timing?

Our first aim was to investigate the relationship between moult timing and age, reproductive status, census year and parasite load. Moult statuses in July and November censuses were modelled as binomial response variables in separate models; all binomial models in our analyses used the threshold family with a probit link function and residual variance fixed at one (Albert and Chib 1993; Hadfield 2010, 2024). In the July census models, females that had moulted half or all of their coat by the census date were defined as 1, while those still with more than half winter coat were defined as 0. In the November census models, females that had moulted half or all of their coat by the census date were defined as 1, whereas those still with more than half summer coat were defined as 0. We ran two models to explore the predictors of moult timing for each moult census: one using the full timeframe available for each census to explore variables recorded across the entire duration of the project, and a second including the parasite data which falls over a shorter timeframe.

July 1st moult status: Across the full study timeframe, we wanted to understand the relationships between reproductive status, individual age and moult timing, in addition to considering any temporal trends in moult (*n* = 3599 obs). There was potential for the condition of females in summer to be impacted by their reproductive status across two reproductive cycles: the previous year and the current. Therefore, we included the previous year's reproductive status, which included the categories outlined above, and their fecundity in the year of the moult census, i.e., whether they gave birth to a calf during the year of the moult census. We also included an interaction between the previous year's reproductive status and fecundity in the same year. We included age and age^2^ as continuous fixed effects, allowing for a non‐linear trend with age as observed in a range of other red deer traits (Nussey et al. 2006). Census year was also added as a continuous fixed effect to determine if there has been change over time. Random terms of year and female ID were also included, controlling for pseudoreplication and allowing us to estimate variances among individuals and years in moult timing. The same model structure was used to estimate the effect of parasite burden on moult status using the shorter timeframe (*n* = 828 obs), but including the preceding April strongyle FEC as an additional fixed effect.

November 1st moult status: Analyses of the predictors of November moult status used the same variables as models of July moult status, only differing in the reproductive status variable used and excluding fecundity (*n* = 5570 obs). When moulting into winter coat, the reproductive status most relevant for condition will depend on the reproduction of the individual in the current year, reflecting whether or not they have been lactating through the summer. To account for this the following categories were included: Immature, Milk (calf alive on 1st Oct; Winter Yeld is not distinguishable from milk in autumn), Summer Yeld and True Yeld. The same November moult status model structure was used to estimate any effect of parasite burden with the addition of the August and November FECs as fixed effects (*n* = 701 obs). Both months were included in the same model to estimate the effect of one season while controlling for the other.

### 2.4 Aim 2: What Does Moult Timing Predict?

Next we examined whether the relationships between moult status and relevant traits were consistent with the hypothesis that the timing of moult reflects individual condition. The traits considered were female survival and the survival of her calf through the winter following the moult censuses, with moult relating to that of the female/mother, in addition to fecundity in the upcoming reproductive cycle and calf birth weight for those that conceived. All models included July moult status and November moult status as three‐level categorical fixed effects; we primarily discuss results comparing the moult statuses of ‘summer’ and ‘winter’, with the results for ‘half’ illustrated in figures.

#### 2.4.1 Female Survival

Resighting rates of females in the population are extremely high (probability > 0.95; Moyes et al. 2006), allowing robust estimates of annual survival. Annual survival was modelled as a binomial GLMM including fixed effects for the July and November moult statuses from the year for which survival was determined (*n* = 2945 obs). July moult status and November moult status were included as fixed effects in addition to reproductive status, female age and age^2^ and year. Reproductive status reflected parental care during the year in which survival was measured and included all categories. The random terms only included year because when female ID was included the model would not converge. Whilst individuals can differ in their survival, as they only die once and survival decreases with age, it can be a difficult variance to estimate. Deaths by shooting were removed from the dataset.

#### 2.4.2 Offspring Survival

The first‐year survival of a calf born in the same year as the mother's moult censuses was modelled using a binomial GLMM (*n* = 1056 obs). The July and November moult statuses of the mother during the offspring's first year were included as fixed effects, to examine how the mother's putative condition was associated with her calf's survival. The fixed effects also included year, mother age and age^2^, the calf's birth date and birth weight and random terms of year and mother ID. Calves that were shot were removed from the dataset.

#### 2.4.3 Future Fecundity

The fecundity of the female in the reproductive cycle following the moult censuses (conception having occurred in the autumn prior to the November moult census) was modelled in a binomial GLMM (*n* = 2818 obs). The model used the same fixed effects as the female survival model, with reproductive status representing the female's parental care during the reproductive cycle prior to the one in which fecundity was measured. Female ID and year were included as random effects.

Birth weight of future calf: Offspring birth weight was modelled using a linear mixed model (LMM) in relation to the moult statuses of the mother over the previous year to consider how moult timing reflects the condition of the female in the run up to conception and moving into winter and gestation (*n* = 1335 obs). The fixed effects and random terms in this model were consistent with the future fecundity model, with the addition of the calf's birth date and sex in the fixed effects as in previous analyses of calf birth weight (Froy et al. 2019; Macphie et al. 2025; Moyes et al. 2011).

### 2.5 Aim 3: Does Moulting Covary With Other Phenological Traits?

We used a multivariate GLMM with four response variables to estimate the covariances between calving dates and July and November moult statuses. The response variables included calving dates (*n* = 2033 obs) and July moult statuses (*n* = 3319 obs) within the same year, occurring close in time, plus the November moult statuses from censuses both before (*n* = 3422 obs) and after the calving event (*n* = 3422 obs). By including the preceding and successive winter moult, we could consider how the temporal pattern of deer condition varies across a 12‐month period and how this connects the timing of events occurring at different points in time. The correlation between the individual‐level effects for November in one year and the following will equal 1, as both are estimating the same coefficient; we do not need to estimate this correlation, but it is produced as part of estimating the individual‐level correlations across the other response pairs we are interested in. Similarly, the individual‐level correlations will be consistent between a given response (calving date or July moult status) and each year's November moult statuses.

The three moult status response variables were modelled as in the full timeframe models when exploring predictors of moult timing in Aim 1 (Table S1). Birth date was modelled as a trait of the mother with a Gaussian distribution. The birth date response included year, female age and age^2^ and the reproductive status of the female over the previous year as fixed effects, with year and female ID as random terms, as is standard for birth date analyses (Froy et al. 2019; Macphie et al. 2025). Different coefficients were estimated for each fixed effect for each response variable. Covariances were estimated among the year, female ID and residual terms for each pair of response variables, which were interpreted as correlations as described in Houslay and Wilson (2017).

In all models, continuous fixed effects were centred. We used the 95% credible intervals (CIs) to interpret our results: fixed effects and covariances/correlations were considered significant if the CIs did not include zero and variance estimates were considered significant if the distribution was removed from zero (as negative estimates are not possible). Coefficients are given on the link scale unless stated otherwise. Throughout this study, we have interpreted a higher probability of moulting by the census date as earlier moult timing and *vice versa*. We aimed to achieve effective sample sizes of over 1000 for focal parameters. With the exception of the multivariate model run for Aim 3, all models were run for 100,000 iterations with a burnin of 7000 and thinning of 75. The multivariate model was run for 450,000 iterations with a burnin of 50,000 and thinning of 250. We used parameter‐expanded priors for random effect (co)variances with marginal variance priors of scaled F_1,1_ with a scale of 100. The residual variance used an inverse gamma distribution with shape = scale = 0.002, except when the response variable was binomial and the residual variance was fixed at one. For all fixed effects default flat priors were used. To visualise our results we used the predict.MCMCglmm function from the MCMCglmm package (Hadfield 2010) and the ggplot2 package (Wickham 2016).

## 3 Results

### 3.1 What Predicts Moult Timing?

#### 3.1.1 Age

Our models show an association between age and moult status in both the July and November censuses. The linear and quadratic age effects were significant and negative in both models: July census linear age coefficient posterior mean [95% CI] = −0.06 [−0.10, −0.02] and quadratic age coefficient = −0.01, [−0.02, −0.01], November census linear age coefficient = −0.12, [−0.15, −0.10] (95% CI) and quadratic age coefficient = −0.010, [−0.014, −0.006]. Negative estimates for both the linear and quadratic age coefficients show that there is a peaked trend in the probability of moulting by the census with age, with the maximum likelihood falling at a lower value than the data was centred on. The pattern is clear in Figure 3, where the probability of moulting is highest in the early years of life for both censuses and then a nonlinear decline with age.

**FIGURE 3:**
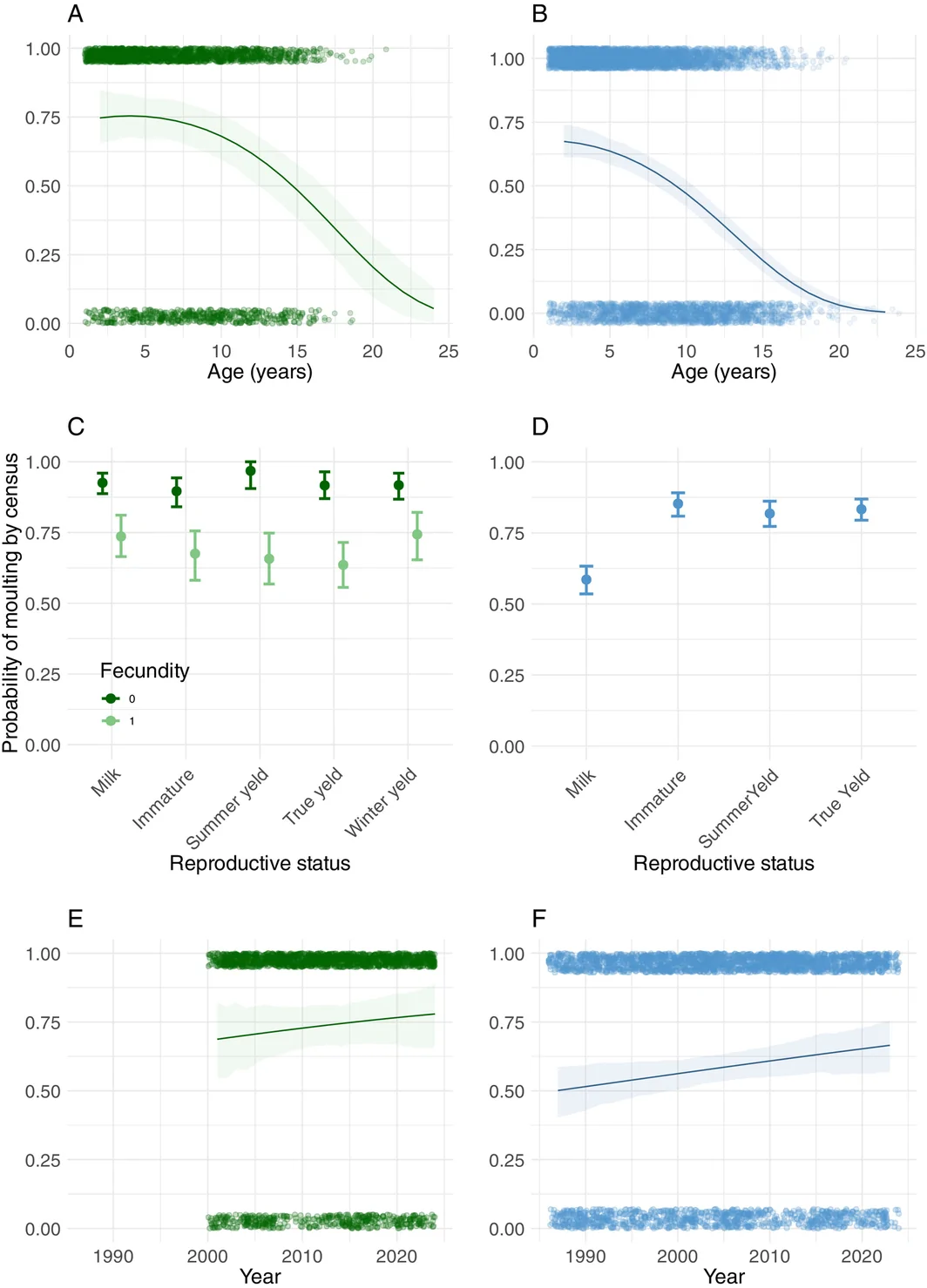
Posterior mean and 95% credible intervals for the probability of moulting by census date relative to age (A, B), reproductive status (C, D) and year (E, F). Left side plots show the July moult status prediction, while right side plots show the November moult status prediction. For the continuous variables, points show the jittered moult census data. Reproductive status differed between the two moult censuses: (C) July moult status was relative to reproductive status in the previous reproductive cycle, fecundity in the current year and their interaction; (D) November moult status was relative to reproductive status in the current year. July moult change has been monitored over a shorter timeframe, as illustrated in (E).

#### 3.1.2 Reproductive Status

Females were significantly less likely to moult by the July census if they had a calf in the same year (−1.35, [−1.65, −1.10]). No differences in July moult status were identified in association with the previous year's reproductive status and no interaction was identified between the previous year's reproductive status and fecundity in the same year (Figure 3C).

The November moult status varied with reproductive status in the year of the moult. Milk females had a lower probability of moulting by the November census compared to females in all other reproductive status categories (Figure 3D). Specifically, the females identified as Immature (1.29, [1.09, 1.50]), Summer Yeld (1.08, [0.87, 1.26]), or True Yeld (1.16, [1.03, 1.28]) were all more likely to moult by the November census than milk hinds (all figures on the link scale).

#### 3.1.3 Census Year

The probability of a female moulting into their winter coat by the November census significantly increased over time (0.02, [0.00, 0.04]; Figure 3F). For the July census, covering a shorter span of years i.e., 24 versus 37, the year slope credible intervals overlapped zero, however, the point estimate was positive and similar to the November census (0.03, [−0.03, 0.08]; Figure 3E).

#### 3.1.4 Variances at the Individual and Year Level

The probability a female moulted by each census varied significantly at the year and individual level. In the models for both the July and November censuses, the impact of female ID was more pronounced than the interannual (year) variation (Figure S1).

#### 3.1.5 Parasites

In the shorter time series of strongyle FEC data, FEC was associated with the probability of moulting by both the July and November censuses. Females were less likely to moult by the July census if they had a high strongyle egg count in April (−0.01, [−0.02, −0.01]) (Figure 4A). Similarly, a female was less likely to moult by the November census if she had a high strongyle count in August (−0.04, [−0.06, −0.02]; Figure 4B) or November (−0.06, [−0.12, −0.01]; Figure 4C).

**FIGURE 4:**
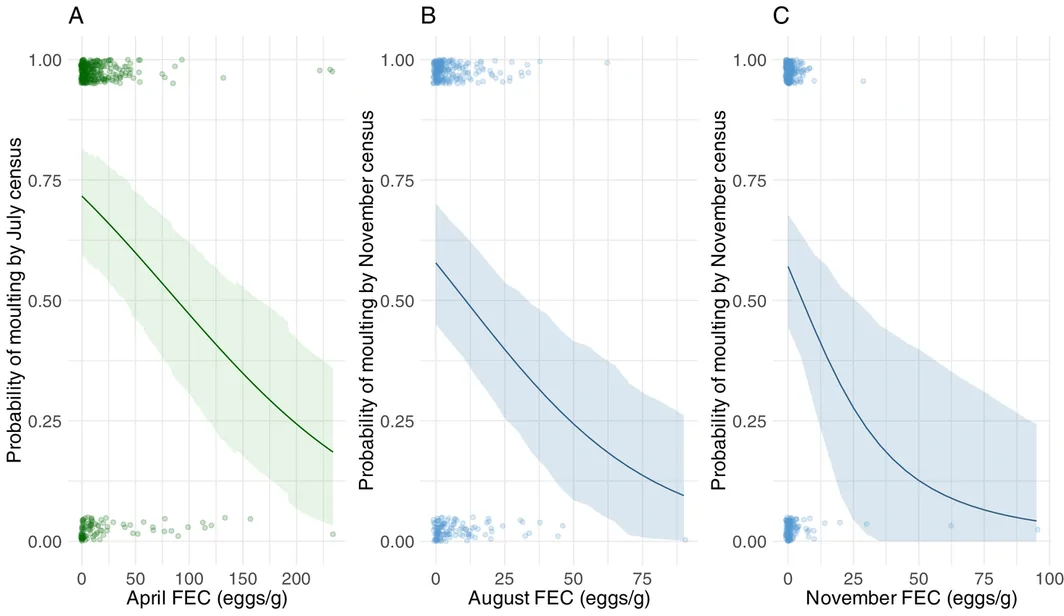
Posterior mean and 95% credible intervals for the probability of moulting by the moult census relative to strongyle faecal egg count. Plot A depicts spring (April) strongyle count as a predictor of moult status at the July moult census. Plot B depicts summer (August) and Plot C depicts autumn (November) strongyle count as predictors of moult status at the November moult census. The points represent the moult census data.

### 3.2 What Does Moult Timing Predict?

#### 3.2.1 Female Survival

A female's moult status in July was not associated with survival through the following year (−0.21, [−0.44, 0.11]; Figure 5A). However, November moult status was a significant predictor of female annual survival, whilst accounting for the effect of age, with females that had moulted by the census being more likely to survive (−0.61, [−0.86, −0.36]; Figure 5A).

**FIGURE 5:**
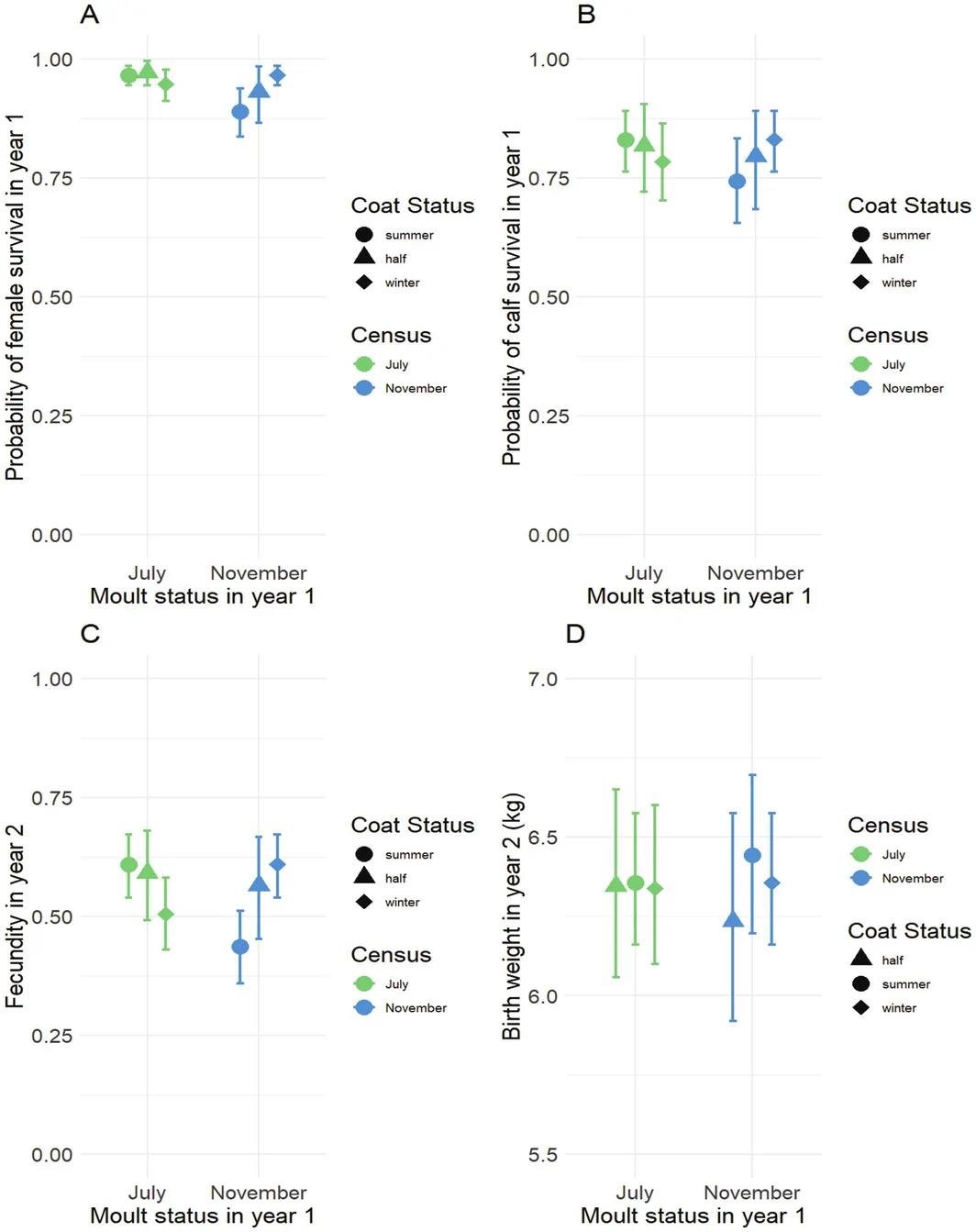
Moult status as a predictor of female survival (A), calf survival (B), fecundity (C) and birth weight (D). (A) Probability a female will survive the year (ending 1st May) relative to their moult status at the July and November moult censuses in the same year. (B) Probability a calf will survive to the end of their first year relative to the mother's moult status at the July and November moult census in the same year. (C) Fecundity relating to conception in the year of the moult censuses relative to the female's moult status at the July and November moult censuses. (D) Predicted birth weight of a calf relative to the female's moult status at the July and November moult census in the year of conception. Points and errorbars show the posterior mean and 95% credible intervals.

#### 3.2.2 Calf Survival

We found an association between the November moult status of a mother and her calf's survival over winter, with calves of females that had moulted by the November census being more likely to survive than if their mother had not moulted (−0.47, [−0.78, −0.16]; Figure 5B). There was no clear effect of July moult status on calf survival (−0.25, [−0.59, 0.06]; Figure 5B).

#### 3.2.3 Future Fecundity and Future Calf Birth Weight

The moult status of females in July and November was both predictive of fecundity. Those that had moulted by the censuses were more likely to have a calf the following summer than females that had not; the point estimates suggest a stronger association with the November census than the July census (July = −0.32, [−0.49, −0.16]; November = −0.54, [−0.71, −0.38]; Figure 5C). When females did have a calf, we found no clear effect of July or November moult status on the future calf's birth weight (July = −0.02, [−0.23, 0.17]; November = 0.08, [−0.09, 0.27]; Figure 5D).

### 3.3 Does Moulting Covary With Other Phenological Traits?

The multivariate model examined the relationship between calving dates, July moult statuses and the November moult statuses from both the same year as the calving dates and July moult and the year before, with the November moult census of the year before falling shortly after the rut when calves born the following summer are conceived (Figure 6). Among years, we found significant correlations between November moult status and calving dates the following year (−0.60 [−0.88, −0.28]) and between July moult status and the November moult status occurring later within the same year (0.50 [0.19, 0.82]). These correlations suggest that in years where the deer on average moult earlier in summer they also moult earlier in the following autumn, and when they moult earlier in the autumn they on average calve earlier the following spring. Among average individual responses across years, we found a positive correlation between average moult status in the November moult census and July moult census (0.48 [0.37, 0.59]), suggesting that individuals consistently moult either earlier or later during both summer and winter moulting events. At the residual level, we found significant correlations between winter moult status and calving date in the following year (−0.12 [−0.19, −0.02]), calving date and summer moult status (0.32 [0.24, 0.40]) and summer moult status and the following winter moult status within the same year (0.34 [0.22, 0.45]). These correlations suggest that in years where individuals moult earlier than expected (for that individual in that year) in relation to the November moult census, they are also likely to calve earlier than expected the following summer. When they calve earlier than expected they are less likely to have moulted by the July moult census that falls shortly after calving, and if they were less likely than expected to moult by the July moult census then they were also less likely to have moulted by the November moult census occurring later on within the same year.

**FIGURE 6:**
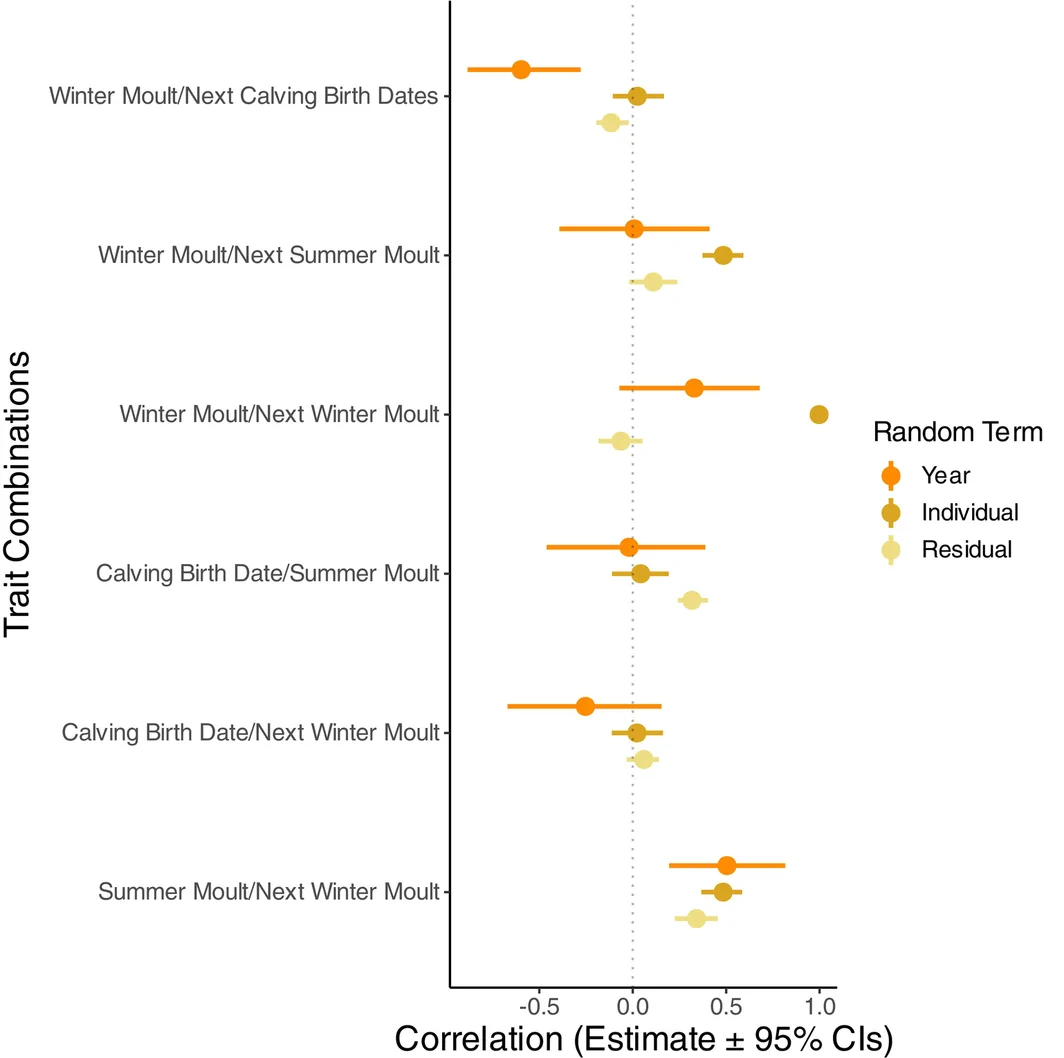
Among individual, year and residual correlations across calving dates, November moult statuses from the year preceding the calving dates and July and November moult statuses of the same year as the calving dates; calculated from the covariance matrices of a four‐response multivariate model.

## 4 Discussion

Our results strongly support the notion that moult timing reflects individual condition in red deer living in the food‐limited environment of north west Scotland. Later moult is associated with multiple variables known to correspond with lowered individual condition, namely ageing, parasite load, pregnancy and calf rearing. Specifically, moult timing at both July and November censuses showed a classic senescent decline with age (Figure 3), July moult is strikingly later in females that have just given birth to a calf (Figure 3C) and November moult is later in females that have lactated to a calf over the summer (Figure 3D), consistent with previous findings in other mammals (Beltran et al. 2019; Déry et al. 2019). In a shorter time series, higher strongyle FECs were associated with later moulting in both July and November (Figure 4).

Similarly, moulting is predictive of several downstream life history events which are likely to be driven by condition (Figure 5). Specifically, the survival of a female and of her current calf (if she had one) was both greater if she had moulted by November census, and if she survived, her future fecundity was higher if she had moulted by both the July and November censuses. On the other hand, the future calf's birth weight was not predicted by moult timing. The birth weight result is consistent with previous research in this study system which has found that birth weight is highly responsive to spring temperature during gestation, presumably acting through plant growth (Macphie et al. 2025). Spring environmental conditions are expected to alter the condition of the mother, uncoupling the maternal condition effects on birth weight from the condition of the mother at the preceding moult censuses.

Moulting advanced over time at the November census and the July moult showed a similar slope, though not statistically significant, which may be due to the shorter time series (Figure 3). Earlier moult timing may reflect an increase in the condition of the individuals over time. A trend towards earlier timing is consistent with several other phenological traits in the population including oestrus, calving, antler casting and cleaning and rut start and end dates (Moyes et al. 2011). While it is tempting to suggest these traits are responding to improving forage under climate change, we have failed to demonstrate a sufficient response to either temperature or measures of available vegetation to explain the change in calving date (Macphie et al. 2025; Macphie et al. 2026). There is also some evidence that the population may be evolving to earlier calving dates in response to selection (Bonnet et al. 2019). Nevertheless, there is no individual‐level correlation between either July or November moult statuses and calving dates (Figure 6) or genetic covariance between calving date and winter moult (Clements et al. 2011), suggesting that any potential genetic change underlying the trend in calving dates would be distinct from the specific processes underlying the trend in moult timing.

We identified substantial variance in summer and winter moult timing at both the individual and year‐level, with estimates suggesting moult timing varies more among individuals than years (Figure S1). In a trait that reflects condition, we expect year effects to reflect annual variation in the environmental conditions experienced by the deer, such as weather and population density, which influence forage availability and thermal stress. The individual‐level variance indicates that individuals are consistently early or late to moult across their lifetimes. We also identified individual‐level correlations across moult events (Figure 6), suggesting that individuals show consistency not just in whether they are early or late to moult within each seasonal moult, but also between the two moult events. The individual‐level variance and covariance among moult events could result from permanent environment effects arising from conditions in early life (Kruuk et al. 1999; Pigeon et al. 2017), or it could be genetic in origin. Permanent environment effects have been documented in a number of adult traits in the study population; for example, high birth weights following warm springs are associated with high lifetime breeding success (Albon et al. 1987; Kruuk et al. 1999). The reported variances are consistent with previous work that identified significant variance among years and individuals in the timing of the moult into winter coat, estimated in a different way to here (Clements et al. 2011). Using an animal model, these authors were able to partition the individual effects into permanent environment and additive genetic variance components, resulting in a heritability of timing of 0.26 (SE 0.06), indicating that some of the individual variance detected in our study is likely genetic in origin.

We have also assessed the year‐ and residual‐level covariances among July moult, November moult and calving dates (Figure 6), which may reflect temporal connections between phenological events associated with individual condition. The year and residual correlations will reflect general patterns among individuals and their deviations from this respectively. We identified a positive relationship between November moult phenology and the birth dates of subsequent calves at the year‐ and residual‐level (Figure 6; a negative coefficient here indicates a positive phenological association), consistent with the hypothesis that individual condition in the autumn influences when deer come into oestrus and conceive, and that conception timing is a large determinant of calving date. Condition at this time is expected to be associated with forage availability throughout the summer and into the autumn, prior to the rut and moult into winter coat (Jeong et al. 2024; Loe et al. 2005; Macphie et al. 2025). We also see positive correlations at the year‐ and residual‐level between July moult status and the following November, but not between November moult status and the following July moult status. This suggests a strong association between the timing of moult events within a year, but not across calendar years. At the juncture between calving dates and July moult status, we see earlier calving dates associated with later moult timing, possibly indicating a trade‐off between moult and reproduction (Constant et al. 2024; Déry et al. 2019; Dietz et al. 2013). Taken together, the temporal distribution of the correlations identified suggests the summer period is a time when condition is to some extent reset. We suspect the seasonal cycle in abiotic conditions and annual plant growing season heavily influence this, with increasingly favourable conditions and the onset of the growing season in spring influencing individual condition through the summer and into the following winter when more challenging conditions return, before the new year's seasonal cycle again provides a boost to condition, somewhat uncoupling the condition of individuals from year to year.

We have shown that in a species in which moult is easily assessed in large numbers of individuals observable in open habitats, it is an indicator of condition which predicts the vital rates of female fecundity and the survival of females and their calves overwinter. This means moult is potentially a valuable tool in deer management. Managers wishing to monitor the condition of the deer population in their area could conduct annual surveys on fixed dates to determine the relative health of the population in each year. The two dates selected for our moult censuses, July 1st and November 1st, are appropriate for red deer in north west Scotland at this time, although if the trend for earlier phenology events continues, the appropriate dates may change. Management plans could then be made based not just on numbers, but also on the state of the population. For example, as survival and fecundity will be higher in years where condition is higher, it may be preferable to cull more heavily. Similarly, if the association between moult timing and condition is similar across other mammalian species of conservation concern, monitoring moult across years could provide valuable information on the health of the population via an observational, non‐invasive method.

Moult could also be used in individual cull decisions. The census date November 1st falls within the currently allowed shooting season for red deer females in the UK, so managers could also use the moult status of individuals to select individuals to be culled. For example, if the motivation for culling is on welfare grounds, to prevent overwinter mortality from natural causes, a stalker might select individuals still in summer coat in November who are likely older and in lower condition. Alternatively, if the motivation is to reduce fecundity, they might selectively shoot individuals that are already in winter coat, as those are most likely to calve the following year.

In conclusion, we have shown that in red deer, the timing of moult is a non‐invasive measure of condition. Moult is later after individuals have invested in costly activities such as lactation or suffered parasite infection, and earlier moult predicts subsequent vital rates. As indicated by moult timing, individuals can recover condition over the summer, but individuals also show repeatability in the timing of moult across moult events throughout their life. Like other phenological traits, moult is getting earlier over time, which could reflect an increase in condition, among other things. As a trait that is plastic, has a genetic component and is associated with both reproduction and survival, there is great value in advancing our understanding of moult and its associations under ongoing environmental change. Given the state of moult of an individual is easily observed, it has potential utility in population management and conservation.

## Author Contributions

**Devyn J. Simeoni:** formal analysis (equal), visualization (lead), writing – original draft (equal), writing – review and editing (equal). **Kirsty H. Macphie:** conceptualization (equal), formal analysis (equal), supervision (supporting), visualization (supporting), writing – original draft (equal), writing – review and editing (equal). **Fiona E. Guinness:** conceptualization (equal), data curation (equal), methodology (equal), writing – review and editing (supporting). **Sean J. Morris:** data curation (equal), writing – review and editing (supporting). **Alison Morris:** data curation (equal), writing – review and editing (supporting). **Ziyi Wang:** formal analysis (supporting), writing – review and editing (supporting). **Tim H. Clutton‐Brock:** data curation (equal), funding acquisition (equal), methodology (equal), writing – review and editing (supporting). **Josephine M. Pemberton:** conceptualization (equal), data curation (equal), funding acquisition (equal), methodology (equal), supervision (lead), writing – original draft (equal), writing – review and editing (equal).

## Funding

This work was supported by the Natural Environment Research Council and European Research Council.

## Ethics Statement

Current project licence PP4166464 under the Animals (Scientific Procedures) Act 1985.

## Conflicts of Interest

The authors declare no conflicts of interest.

## Supporting information


**Table S1:** Models run, including, response, fixed and random terms and sample sizes.
**Figure S1:** Posterior distributions for the variance components of moult status at July and November moult censuses.

## Data Availability

Data and code are available at https://doi.org/10.5281/zenodo.23335445.
