# Supplementary material for: Moult Timing as a Measure of Condition in a Northern Hemisphere Ungulate
Source: Ecol Evol. 2026 Nov 1;16(11):e74493. doi: 10.1002/ece3.74493 (PMC13724253; doi:10.1002/ece3.74493)
Supplement: Supplementary file 1 [file ECE3-16-e74493-s001.docx]

**Supporting information for ‘Moult timing as a measure of condition in a northern hemisphere ungulate’**

*Table S1. Models run, including, response, fixed and random terms and sample sizes.*

| **Predictors of moult status** | | | | | |
| --- | --- | --- | --- | --- | --- |
| **Model Type** | **Response** | **Fixed effects** | **Random terms** | **N. observations** | **N. females** |
| Binomial generalized linear mixed model | July moult status (0/1) | Reproductive status (previous year)  Fecundity (same year)  Year  Age + age^2^ | Year  Female ID | 3599 | 656 |
| Binomial generalized linear mixed model | July moult status (0/1) | Reproductive status (previous year)  Fecundity (same year)  Year  Age + age^2^  April strongyle FEC | Year  Female ID | 828 | 261 |
| Binomial generalized linear mixed model | November moult status (0/1) | Reproductive status (same year)  Year  Age + age^2^ | Year  Female ID | 5570 | 903 |
| Binomial generalized linear mixed model | November moult status (0/1) | Reproductive status (same year)  Year  Age + age^2^  August or November strongyle FEC | Year  Female ID | 710 | 204 |
| **Moult status as a predictor** | | | | | |
| **Model Type** | **Response** | **Fixed effects** | **Random terms** | **N. observations** | **N. females** |
| Binomial generalized linear mixed model | Female survival (0/1) | July and November moult status  (Categorical: summer, half, winter)  Reproductive status (next year)  Year  Age + age^2^ | Year | 2945 | 547 |
| Binomial generalized linear mixed model | Calf survival (0/1) | July and November moult status  (Categorical: summer, half, winter)  Year  Age + age^2^  Calf birth date (same year, ordinal)  Calf birth weight (same year, kg) | Year  Female ID | 1056 | 333 |
| Binomial generalized linear mixed model | Fecundity (0/1) | July and November moult status  (Categorical: summer, half, winter)  Reproductive status (next year)  Year  Age + age^2^ | Year  Female ID | 2818 | 539 |
| Linear mixed model | Birth weight (kg)  (continuous) | July and November moult status  (Categorical: summer, half, winter)  Reproductive status (next year)  Year  Age + age^2^  Calf Birth Date (next year, ordinal)  Calf Sex (Categorical: female, male) | Year  Female ID | 1335 | 407 |
| **Covariances among phenological traits** | | | | | |
| **Model Type** | **Response** | **Fixed effects** | **Random terms** | **N. observations** | **N. females** |
| Multivariate generalized linear mixed model | November moult status (0/1)  Next calving date (continuous)  Next July moult status (0/1)  Next November moult status (0/1) | *November moult status:*  Reproductive status (same year)  *Next calving date:*  Reproductive status (previous year)  *Next July moult status:*  Reproductive status (previous year)  Fecundity (same year)  *Next November moult status:*  Reproductive status (same year)  *All response variables:*  Year  Age + age^2^ | Year VCV across responses  Female ID VCV across responses  Residual VCV across responses  (Variance fixed at one for binary traits) | *November moult status:*  3422  *Next calving date:*  2033  *Next July moult status:*  3319  *Next November moult status:*  3422 | 508 |

**Figure S1.** Posterior distributions for the variance components of moult status at July and November moult censuses. The green curves illustrate the female identity ID and year variance estimates for the July moult census. The blue curves illustrate the female identity and year variance estimates for the November moult census. An orange dashed line is marked at zero to illustrate where each posterior distribution sits relative to zero.
